# Protective role of nitric oxide donors on endothelium in ischemia-reperfusion injury: a meta-analysis of randomized controlled trials

**DOI:** 10.1186/s12871-023-02117-w

**Published:** 2023-05-31

**Authors:** Chaoxun Dou, Xue Han, Hanbin Xie, Haofeng Liao, Xue Xiao, Ziyan Huang, Gangjian Luo, Xinmin Zhang, Weifeng Yao

**Affiliations:** 1 Department of Anesthesiology, The third Affiliated hospital of Sun Yat-sen University, Guangzhou, 510630 China; 2 Department of Anesthesiology, Sun Yat-sen Memorial Hospital, Sun Yat-sen University, Guangzhou, 510120 China; 3 Department of Anesthesiology, The First Hospital of Jilin University, Changchun, 130021 China

**Keywords:** Nitric oxide donors, Ischemia reperfusion, Endothelium, Flow-meditate dilation, Meta-analysis

## Abstract

**Background:**

Decreased bioavailability of nitric oxide (NO) under hypoxic conditions can lead to endothelial dysfunction. NO supplementation may protect endothelial function in ischemia-reperfusion (IR) injury. Therefore, a meta-analysis of randomized controlled trials (RCTs) was performed to verify the protective effect of NO donors on endothelium in IR injury.

**Methods:**

Medline, Embase, Cochrane Library, and Web of Science databases were searched from inception to April 1, 2023. The specific inclusion criteria were as follows: (1) RCTs; (2) trials comparing NO donors with placebo control groups; and (3) trials reporting the effects of these interventions on vascular endothelial functional outcomes in IR injury. Random-effects models were used to assess pooled effect sizes, which were expressed as standardized mean differences (SMD).

**Results:**

Seven studies satisfied the inclusion criteria and consisted of a total of 149 participants. NO donors were protective of endothelial function in IR injury (SMD: − 1.60; 95% confidence interval [CI]: − 2.33, − 0.88, *P* < 0.0001; heterogeneity [I^2^ = 66%, *P* = 0.001]). Results of the subgroup analysis showed the following: absence of protective effect of NO donor use following ischemia on endothelial function in IR injury − 1.78 (95% CI: − 2.50, − 1.07) and loss of protective effect on endothelial function after prolonged NO donor use − 0.89 (95% CI: − 2.06, 0.28).

**Conclusion:**

The short-period use of NO donors before the onset of ischemia can protect endothelial function in IR injury.

**Supplementary Information:**

The online version contains supplementary material available at 10.1186/s12871-023-02117-w.

## Introduction

Ischemia/reperfusion (I/R) is a complex pathophysiological process that leads to organ damage in many clinical conditions (e.g., myocardial infarction, organ transplantation, and cardiac arrest). Concurrently, I/R induces vascular endothelial dysfunction through multiple mechanisms, including cytotoxicity caused by pH changes, oxidative stress caused by overproduction of reactive oxygen species (ROS), and endothelial nitric oxide synthase (eNOS)–nitric oxide (NO) inhibition [1–3]. Endothelial cells have been shown to play a key role in the damage sustained after ischemia and reperfusion [4]. Following the discovery of endogenous (endothelial-derived) NO and the recognition of its importance in the control of vascular homeostasis [5], it was speculated that NO donors might be able to provide IR protection [6]. NO production via NOS is inhibited under hypoxic or anoxic conditions [7, 8]. However, as oxygen is being consumed, many proteins involved in the oxidation process at basal oxygen levels may become reductases to convert nitrite to NO. This shift in biological role often occurs in proteins containing heme or molybdenum pterin cofactors such as hemoglobin, myoglobin, and xanthine oxidase (XO) [9–12]. In hypoxic conditions, NO donors increase the amount of NO in the body independent of the L-arginine-NO synthase pathway, making it an attractive alternative therapy to help improve endothelial dysfunction secondary to NO insufficiency. In animal models, NO donors have been shown to reduce I/R injury [13, 14]. However, exogenous NO donor administration has potentially deleterious effects; for example, NO has been shown to play a key role in apoptosis by reacting with superoxide to form peroxynitrite, inducing tyrosine nitration and deleterious protein changes [15, 16]. There is also evidence that treatment with most organic nitrates at clinical doses impairs responsiveness to stimuli of endothelial-derived NO release [17]. Endothelial dysfunction has been observed in animal studies and in humans during extended nitroglycerin (GTN) and isosorbide mononitrate (ISMN) treatment [18]. These contradictory studies suggest that the dominant role of NO in I/R on endothelial function is unclear. Anderson et al. [19] invented the flow-mediated dilation (FMD) method at the brachial artery, which is presently an established, rapid, accurate, and noninvasive assessment method to measure and reflect endothelial function in subjects.

The aim of this article is to illustrate the effect of NO donors on endothelial function (FMD) in human subjects with I/R injury by systematically analyzing evidence from randomized controlled trials (RCTs).

## Methods

In this systematic review, meta-analyses were performed in strict accordance with the criteria stated in PRISMA. The protocols retrieved for this study were registered on the PROSPERO platform (registration number CRD42022365781).

### Search strategy

We searched the Cochrane Library, Medline, Web of Science, and Embase databases from the establishment of the database until April 1, 2023 using a search strategy consisting of a combination of subject terms and free words. Subject terms included reperfusion injury, nitric oxide donors, endothelium. (The complete search strategy for Medline is in the supplementary materials). We searched relevant local and international clinical research trial registry databases and consulted experts in the field to identify potential unpublished data. We also screened the reference lists of all articles selected for inclusion to identify additional studies for potential inclusion.

### Inclusion and exclusion criteria

We included all human clinical studies in which NO donor agents were administered during I/R. Inclusion criteria were as follows: (1) RCTs; (2) studies in which flow-mediated dilation of the brachial artery was measured by ultrasound, namely the FMD method, to reflect vascular endothelial function; (3) trials comparing NO donors with placebo control groups; and (4) trials reporting the effects of these interventions on vascular endothelial function outcomes in I/R. Exclusion criteria included the following: (1) nonclinical studies; (2) lack of complete FMD data; (3) no available full text; (4) presence of other influences besides NO; (5) reviews, newsletters, or editorials.

### Literature selection and data extraction

Two reviewers (Yao and Dou) independently performed an initial screening for relevance by reviewing titles and abstracts of identified articles for potential eligibility. Following the screening for relevance, the two reviewers compared their results. In cases of discrepancies, a third reviewer (Xie) assessed the abstracts, and a consensus was reached among the three reviewers. All studies that were considered potentially relevant were retrieved, and the complete manuscripts were reviewed for inclusion.

Following the completion of the study selection, the following data was extracted from the included studies: (1) authors and publication date of the study; (2) type of study; (3) study subjects and their health status; (4) drugs used, their doses, etc.; (5) number of people included in the study; (6) time course of NO donor application; (7) FMD baseline and endpoint values; and (8) FMD baseline and endpoint change values if available. When the exact data was not available, and only graphs and bars were available, we approached the authors to obtain unpublished data. If unsuccessful, a digital scale was used to estimate the data in the graph or bar chart, and the original data was derived based on estimation according to the axes [20]. As the main observed outcome of this meta-analysis is the change in FMD from baseline to endpoint after the use of NO donor agent, the change values were extracted directly if they were directly provided in the literature, and where only baseline and endpoint values were available, the change values were calculated according to the method proposed in the *Cochrane Handbook for Systematic Reviews of Interventions* for calculating change values in the meta-analysis and Follman’s theory [21]. The following formula was applied for the calculation of the FMD change values:

**Table:** 

	Baseline	Final	Change
Experimental Intervention	Mean_E,b_,	Mean_E,f_,	Mean_E,change_,
	SD_E,b_	SD_E,f_	SD_E,change_
Control Intervention	Mean_C,b_,	Mean_C,f_,	Mean_C,change_,
	SD_C,b_	SD_C,f_	SD_C,change_

$$\text{Mean}_\text{E,change}=\text{Mean}_\text{E,f}-\text{Mean}_\text{E,b}$$

$$\text{Mean}_\text{C,change}=\text{Mean}_\text{C,f}-\text{Mean}_\text{C,b}$$

$$\mathrm{SD}_\text{E,change}=\sqrt{\text{SDE,b2}}+\text{SDE,f2}-\left(2\times\mathrm{R}\times\text{SDE,b}\times\text{SDC,f}\right)$$

$$\mathrm{SD}_\text{C,change}=\sqrt{\text{SDC,b2}}+\text{SDC,f2}-\left(2\times\mathrm{R}\times\text{SDC,b}\times\text{SDC,f}\right)$$

An estimated correlation value of R = 0.50 was used to provide conservative estimates based on the assumption that this value would minimize the error in the effect size estimates.

### Risk of bias and quality assessment

Two researchers (Yao and Dou) assessed the quality of each study using the Cochrane Risk of Bias tool to assess the following: selection bias (random sequence generation and allocation concealment), performance bias (blinding of participants and researchers to the intervention), detection bias (blinding of outcome assessment), attrition bias (completeness of outcome data, including handling of attrition and data exclusion in analysis), and reporting bias (selective outcome reporting) [22].

### Data synthesis and analysis

After obtaining the FMD change values and standard deviations for each study, data on the number of participants, FMD change values, and standard deviations in the experimental and control groups of each study were entered into the REVMAN software, and a random effects model was used to provide a more conservative estimate of the pooled effect size [23]. The standardized mean difference (SMD) and 95% confidence interval (95% CI) for each included study in this meta-analysis were obtained. The software was also used to calculate the final results of the meta-analysis and their *p*-values, plot a forest plot, and derive the values for heterogeneity between studies in this meta-analysis. Heterogeneity is expressed as I^2^. According to international standards, I^2^ > 50% indicates significant heterogeneity between studies while a value of less than 50% indicates the absence of heterogeneity between studies [24].

Exploring the source of heterogeneity was one of the important purposes of meta-analysis, and we selected the following factors as potential sources of heterogeneity and explored their effects on the outcome (FMD change values) via subgroup analysis: (1) timing of NO donor agent administration (i.e., pre-ischemic treatment or post-ischemic treatment) and (2) duration of NO donor agent administration (i.e., short-term treatment or long-term treatment, based on a 24-h cutoff). We first performed a sensitivity analysis to determine their effect on the stability of the results. Next, we applied subgroup analysis and sequentially divided the included studies into two different subgroups according to potential influencing factors, which were entered into the REVMAN software to derive the final results and heterogeneity for each of the two subgroups. The differences in the results between the two subgroups were observed, and if the differences were statistically different, they indicated that the potential factor was the main influencing element affecting this meta-analysis. Concurrently, based on an observation of the magnitude of heterogeneity among subgroups, if the heterogeneity of one or both of the subgroups disappeared, it further indicated that the potential factor was the main source of heterogeneity.

We also plotted funnel plots to clearly and accurately reflect the presence of bias in this meta-analysis.

## Results

### Study inclusion

**Fig. 1:**
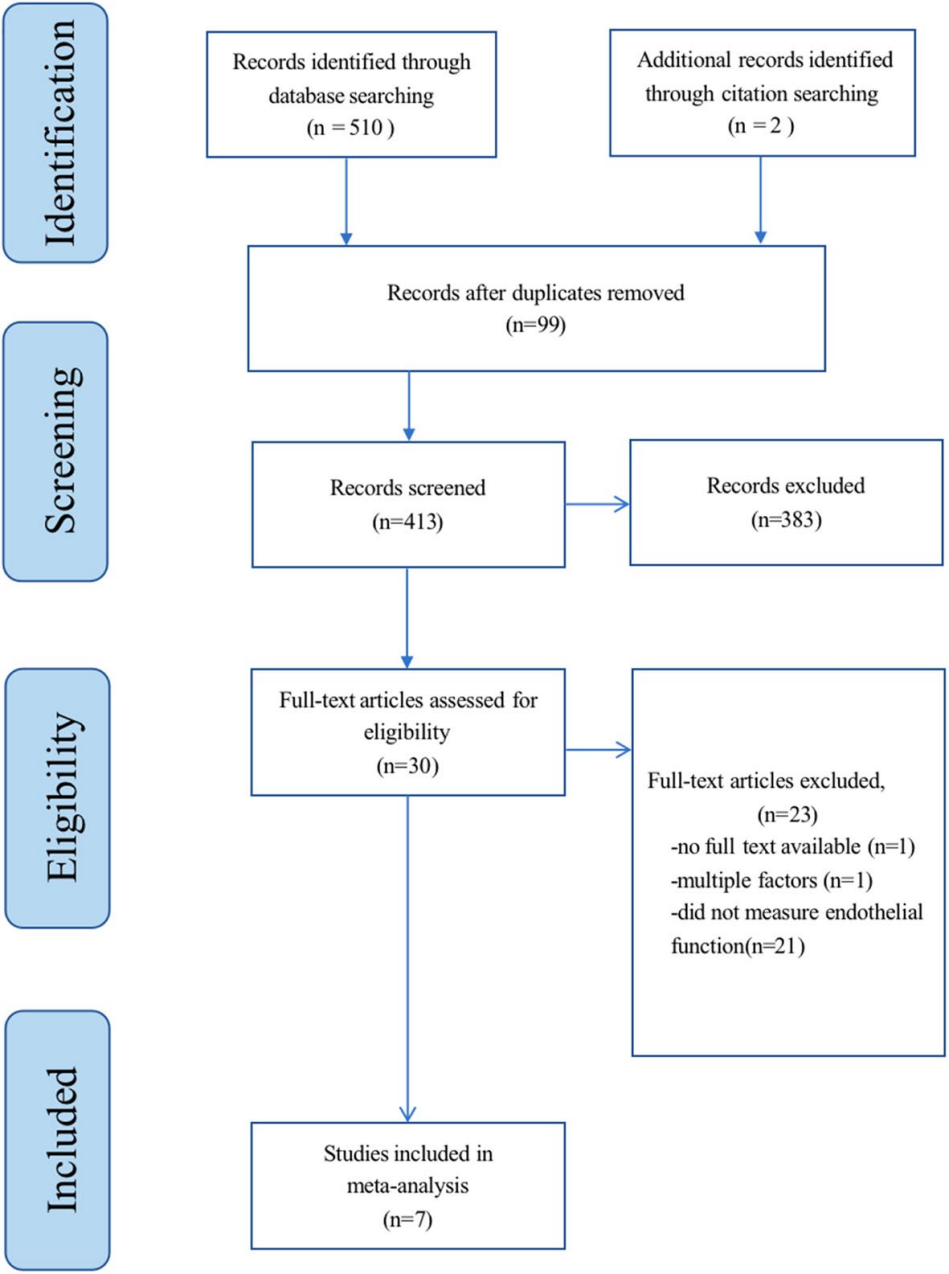
Flowchart of the study selection process in the meta-analysis

A total of 510 articles were found from the search and after removing duplicates (*N* = 99), 411 articles were screened based on the title and abstract, and 383 articles were excluded based on the exclusion criteria. A total of seven articles were selected for further screening, and their complete manuscripts were reviewed, of which two articles were excluded because one of them could not be found in usable full text and the other [25] contained confounding factors (aspirin) in addition to NO, resulting in the inclusion of five studies in the systematic review. We also referred to the references of these five articles and found two more that met the eligibility criteria for the review, which totaled up to seven articles [26–32], of which four articles included two independent clinical studies, thereby increasing the final number of included studies in the meta-analysis to 11. The flowchart of the study selection process is shown in Fig. 1.

### Characteristics of the included studies

**Fig. 2:**
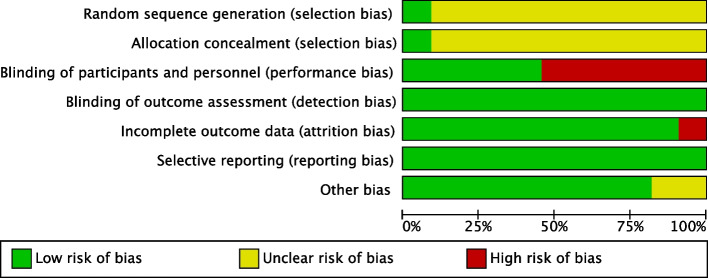
Risk of bias assessment for each included study

The seven studies were published in the last 15 years (2007–2022) and included a total of 149 participants. Six of the studies involved healthy populations and one [25] involved early menopausal women. The number of participants included in each study ranged from 9 to 34. Of the sources of NO, four were from GTN, two from pentaerithrityl tetranitrate (PETN), two from beetroot, and one from nitrite. The choice of placebo for the control group was conducted on a case-by-case basis and included saline, beetroot juice without NO_3_^−^, and no special treatment. Routes of administration included intravenous, oral, and transdermal, with a dosing duration ranging from 20 min to 7 days. The GTN dose was 0.6 mg/h for 2 h/day in all four trials. The PTNE dose was 80 mg/day. Only one study [28] was administered after the onset of ischemia; the remaining studies were administered before I/R. No adverse effects were reported in any of the studies. The quality of the studies indicated an overall low risk of bias. However, only two reported information on random sequence generation and allocation concealment, and the remaining two reported unknown risks. In five of the trials, the researchers were blinded. The risk of bias assessment graph is shown in Fig. 2. The specific detailed characteristics of each study are shown in Table 1.

**Table 1:** Characteristics of the included studies

Study	Year	Study design	Participants	Sample Size	Intervention Drug	Intervention Drug Dosage	Duration	Side Effects
Saverio Dragoni	2007	R,DB,PC	Healthy population	28	GTN, PETN	GTN1.2 mg 2 h; PETN80 mg/d	24 h	Not reported
Tommaso Gori	2007	R,DB,PC	Healthy non-smokers males	17	GTN	0.6 mg/h, 2 h/day	24 h	Not reported
Andrew J. Webb	2008	R,SB,PC	Healthy population	10	Beetroot juice	500 ml	2 h	Not reported
Thomas E	2013	R,SB,PC	Healthy population	10	Nitrite	1 μmol/ml,1.5 ml/min for 20 min	pre, post 20 min	Not reported
Kelsey McLaughlin	2014	R,SB,PC	Healthy population	34	GTN	0.6 mg/h, 2 h/day	24 h, 7 day	Not reported
Y.B. Somani	2022	R,SB,PC	Early postmenopausal women	11	Beetroot juice	140 ml,about nitric acid 9.7 mmol	pre 100 min	Not reported
Monica Lisi	2012	R,SB,PC	Healthy population	20	GTN, PETN	GTN0.6 mg/h, 2 h/day; PETN80 mg/d	pre 7 day	Not reported

*R* randomized, *SB* single-blind, *DB* double-blind, *PC* placebo-controlled, *pre* pre-ischemic, *post* post-ischemic

### Results of meta-analysis

**Fig. 3:**
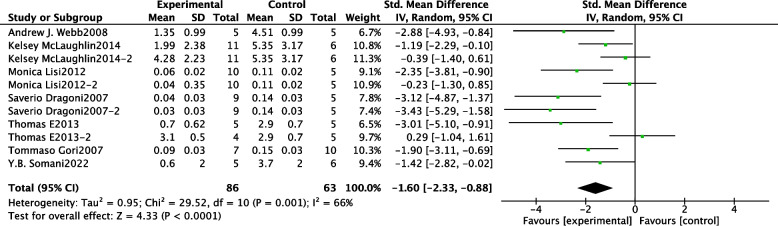
Forest plot of the effect of NO donor intervention on endothelial function in I/R injury

The results of our meta-analysis showed that in 11 experiments, consisting of a total of 149 participants, NO donors had a protective effect on endothelial function in I/R injury (SMD: -1.60; 95% CI: − 2.33, − 0.88, *P* < 0.0001). However, there was significant heterogeneity in the results (I^2^ = 66%, *P* = 0. 001) (See Fig. 3 for specific results). The results of the case-by-case sensitivity analysis have low sensitivity.

**Fig. 4:**
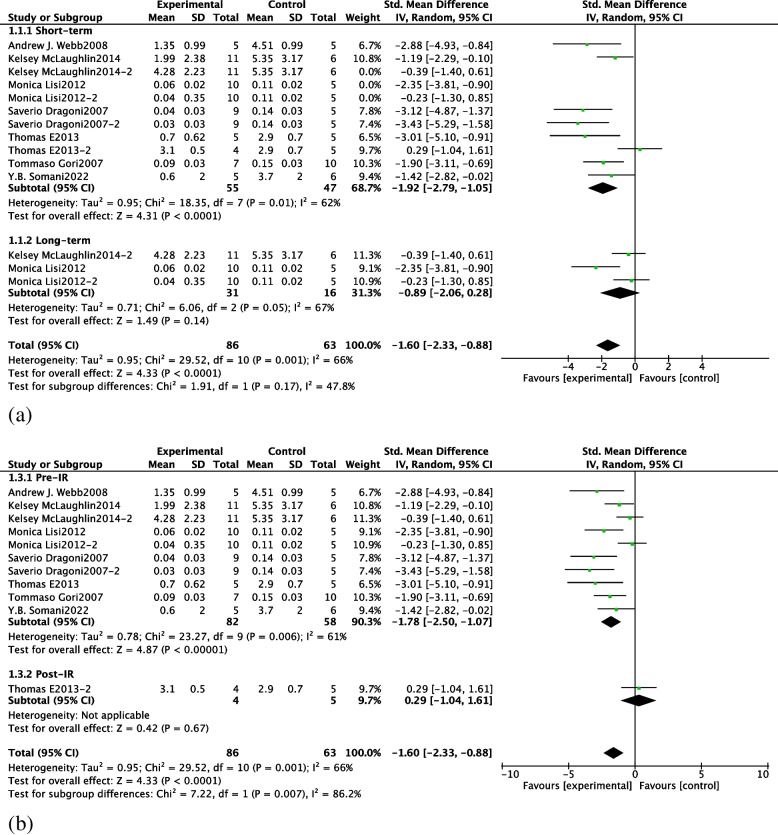
Forest plots for subgroup analysis according to duration of NO donor use (**a**) and timing of NO donor use (**b**). Short-term: short-term use (< 24 h) of NO donor; long-term: long-term (> 24 h) use of NO donor. Pre-I/R: pre-ischemic use of NO donor; post-I/R: post-ischemic use of NO donor

We divided the different subgroups according to the timing of NO donor administration and the duration of NO donor administration, and the results are as follows: (1) Using the duration of NO donor administration as a cutoff, the results of the subgroup analysis were − 0.89 (95% CI: − 2.06, 0.28) for the long-term group and − 1.92 (95% CI: − 2.79, − 1.05) for the short-term group, with no statistically significant difference between the two groups (*P* = 0. 17) (See Fig. 4a for specific results).(2) Using the timing of NO donor administration as a cutoff, the results of the meta-analysis were − 1.78 (95% CI: − 2.50, − 1.07) for the pre-ischemic subgroup and − 1.78 (95% CI: − 2.50, − 1.07) for the post-ischemic subgroup, with statistically significantly different results between the two subgroups (*P* = 0.007) (See Fig. 4b for specific results).

### Publication bias results

**Fig. 5:**
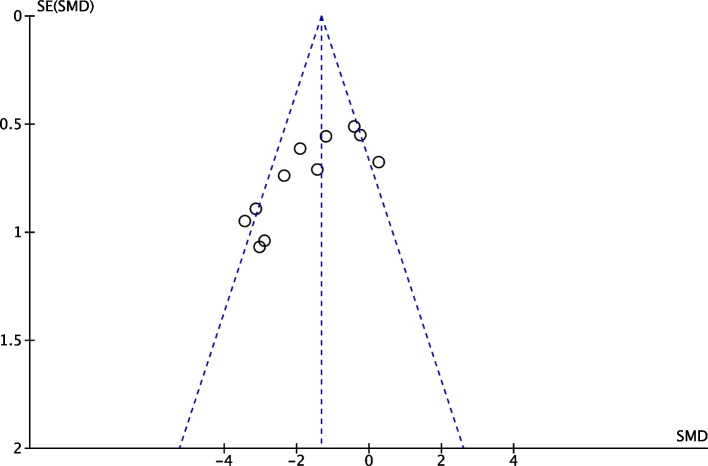
Funnel plot of NO donor effects on endothelial function in I/R injury

The funnel plots were symmetrical, indicating no significant publication bias in the results obtained from the 11 studies. The funnel plots are shown in Fig. 5.

## Discussion

This is the first meta-analysis of the protective effect of NO donors on endothelial function in I/R, and overall, the present study indicates that NO donors have a protective effect on the endothelium in IR injury. However, the timing as well as the duration of NO donor use can affect the final outcome.

The results of the subgroup analysis in this study support the short-time use of NO donors before the onset of ischemia to prevent endothelial dysfunction due to I/R injury. However, all subgroups show significant heterogeneity, so this heterogeneity is likely to be real and may be due to differences in the population, Intervention drugs and Intervention drug dosage included in this study. NO_2_^−^ entering the tissues can be used during hypoxia, or it can be converted to metabolites and stored in the tissues; its effects can be transmitted from the vascular surface to the tissues, thus preventing subsequent injury [33]. Most animal models suggest a beneficial effect on I/R injury when NaNO_2_ is injected prior to ischemia [34]. Under hypoxic conditions, hypoxic signaling reduces eNOS expression and activity. Meanwhile, by interfering with BH4 and L-Arg/ADMA-related activity or expression, it causes eNOS uncoupling, and uncoupled eNOS is an important source of free radicals that can disrupt redox homeostasis and cause oxidative stress. Simultaneously, hypoxia impairs the NO pathway by stimulating endothelial activation to increase oxygen radical production from the mitochondrial respiratory chain and NADPH (Nicotinamide Adenine Dinucleotide Phosphate) oxidase. Relying on exogenous NO supplementation alone at this point does not increase NO bioavailability but may instead increase peroxynitrite production and thus cause further endothelial dysfunction. Prolonged use of NO donors leads to loss of protective effect on endothelial function. This may be due to the development of tolerance after prolonged use of NO donors. After repeated administration, oxidative modifications in ALDH-2 may lead to loss of function of the enzyme, resulting in reduced GTN biotransformation and inhibition of the final effects of pharmacological pretreatment [35, 36]. However, not all nitrates are tolerated, and there is experimental evidence that long-term continuous application of PETN [37] does not lead to the development of tolerance, in part due to the ability of PETN to induce HO-1.

NO is extremely unstable in the body and is usually stored in the body in the form of nitrate or nitrite. In the past, nitrate or nitrite was often considered as inert products of NO oxidative metabolism. However, discoveries in recent years have shown that nitrite can act as a source of NO in vivo, especially under hypoxic conditions. Studies using bactericides to kill the oral microbiome or systemic NOS inhibition have shown that about half of the basal plasma nitrite comes from the reduction of nitrate in saliva and the other half from the oxidation of NO produced primarily by eNOS [38]. Nitrate ingested through the diet is reduced to nitrite by oral flora [39], which forms a pool of nitrite, along with nitrite from the oxidation of NO produced by NOS in the body. The reduction of nitrite to NO in the vascular system can be mediated by a variety of proteins, including heme proteins (heme proteins) [40, 41], XO, carbonic anhydrase [42], heme P450 cytochromes [43], etc. The primary mechanism for the hemodynamic effects of nitrate is thought to be attributed to the activation of the intracellular NO receptor enzyme soluble guanylate cyclase, leading to increased bioavailability of cGMP and activation of cGMP-dependent protein kinase and/or cyclic nucleotidylated ion channels [44]. In addition, S-nitrosylation of key intracellular proteins may reduce mitochondrial L-type Ca2^+^ channel function [45] and inhibit complex I of the electron transport chain, which generates ROS during reperfusion [46, 47]. Together, these theories suggest that the use of NO donor drugs is an effective alternative therapy for “NO-deficient” diseases.

However, there are several limitations to this meta-analysis. Firstly, the number of included studies and the total number of participants were relatively small. There were few high-quality studies on the effects of NO donor agent administration on endothelial function in patients with I/R injury. However, the use of NO donor agents in patients with I/R injury did not result in adverse events, such as hypotension or methemoglobinemia. Our results are important for clinical scientists who wish to further investigate ways to mitigate endothelial dysfunction in I/R injury. Secondly, the NO agents used in the included trials varied in doses and types and had different clinical protocols, leading to a high degree of heterogeneity. Meta-analyses are based on retrospective analytical extrapolation and may be influenced by a variety of factors, such as the inclusiveness of the search strategy for determining eligible studies, assumptions about the consistency of the methods applied across studies, and limited accessibility to data from individual studies. However, our clear delineation of inclusion and exclusion criteria and comprehensive searches of the four major electronic databases and reference lists were likely to minimize bias and increase the representativeness of the results.

## Conclusion

Normal endothelial cell function plays a key role in maintaining vascular homeostasis. Previous studies have confirmed that nitrates improve endothelial function in patients without IR; however, there has been no systematic evaluation of whether NO supplementation improves endothelial function in patients with IR. In the present study, we found that pre-ischemic, short-term application of NO donors protected endothelial function in patients with IR. For endothelial dysfunction in human subjects with I/R injury, administration of NO donor agents may be an effective treatment.

## Supplementary Information

**Additional file 1: Supplemental Material.** Search strategy for Medline.

## Data Availability

The datasets used and/or analysed during the current study are available from the corresponding author on reasonable request.

## Abbreviations

NO: Nitric oxide
CI: Confidence interval
I/R: Ischemia/reperfusion
ROS: Reactive oxygen species
eNOS: Endothelial nitric oxide synthase
GTN: Nitroglycerin
FMD: Flow-mediated dilation
NADPH: Nicotinamide Adenine Dinucleotide Phosphate
PRISMA: Preferred Reporting Items for Systematic Review and Meta-Analysis
RCTs: Randomized controlled trials
SMD: Standardized mean difference
