# Supplementary material for: Protective role of nitric oxide donors on endothelium in ischemia-reperfusion injury: a meta-analysis of randomized controlled trials
Source: BMC Anesthesiol. 2023 May 31;23:189. doi: 10.1186/s12871-023-02117-w (PMC10230692; doi:10.1186/s12871-023-02117-w)
Supplement: Supplementary file 1 — Additional file 1: Supplemental Material. Search strategy for Medline. [file 12871_2023_2117_MOESM1_ESM.docx]

**Supplemental Material:search strategy for Medline**

#### **((((((((((((reperfusion injury[MeSH Terms]) OR (Reperfusion Injuries[Title/Abstract])) OR (Reperfusion Damage[Title/Abstract])) OR (Damage, Reperfusion[Title/Abstract])) OR (Reperfusion Damages[Title/Abstract])) OR (Ischemia-Reperfusion Injury[Title/Abstract])) OR (Ischemia Reperfusion Injury[Title/Abstract])) OR (Injury, Ischemia-Reperfusion[Title/Abstract])) OR (Injury, Ischemia Reperfusion[Title/Abstract])) OR (Injury, Ischemia Reperfusion[Title/Abstract])) OR (Injury, Reperfusion[Title/Abstract])) AND (((endothelium[MeSH Terms]) OR (Endotheliums[Title/Abstract])) OR ((((((nitric oxide donors[MeSH Terms]) OR (Donors, Nitric Oxide[Title/Abstract])) OR (Oxide Donors, Nitric[Title/Abstract])) OR (Nitric Oxide Donor[Title/Abstract])) OR (Donor, Nitric Oxide[Title/Abstract])) OR (Oxide Donor, Nitric[Title/Abstract])))) AND (randomized controlled trial)**
